# Supplementary material for: A 2.2 Å cryoEM structure of a quinol-dependent NO Reductase shows close similarity to respiratory oxidases
Source: Nat Commun. 2023 Jun 9;14:3416. doi: 10.1038/s41467-023-39140-x (PMC10256718; doi:10.1038/s41467-023-39140-x)
Supplement: Supplementary file 1 — Supplementary information [file 41467_2023_39140_MOESM1_ESM.pdf]

## Supplementary information

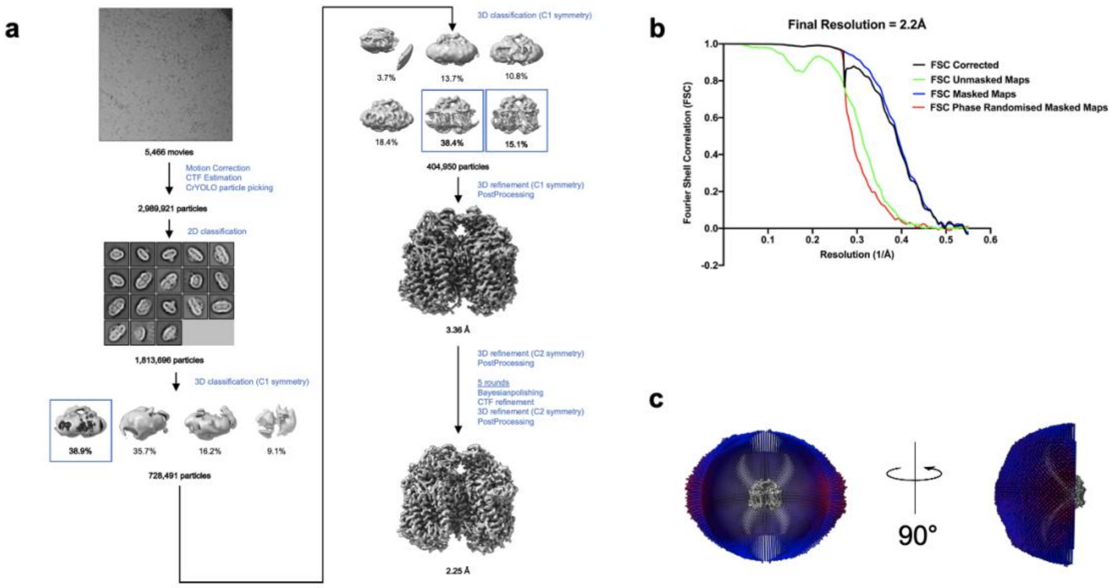


**Supplementary figure 1: CryoEM analysis of quinol-dependent Nitric Oxide Reductase (qNOR) from *Alcaligenes xylosoxidans.* (A)** Cryo-EM image processing workflow performed in RELION. Example micrograph, 2D classes, 3D classes and refined reconstructions are highlighted. **(B)** FSC curve for the final map. Final resolution was calculated using the gold-standard FSC cut-off of 0.143. **(C)** Euler angle distribution of refined particles.

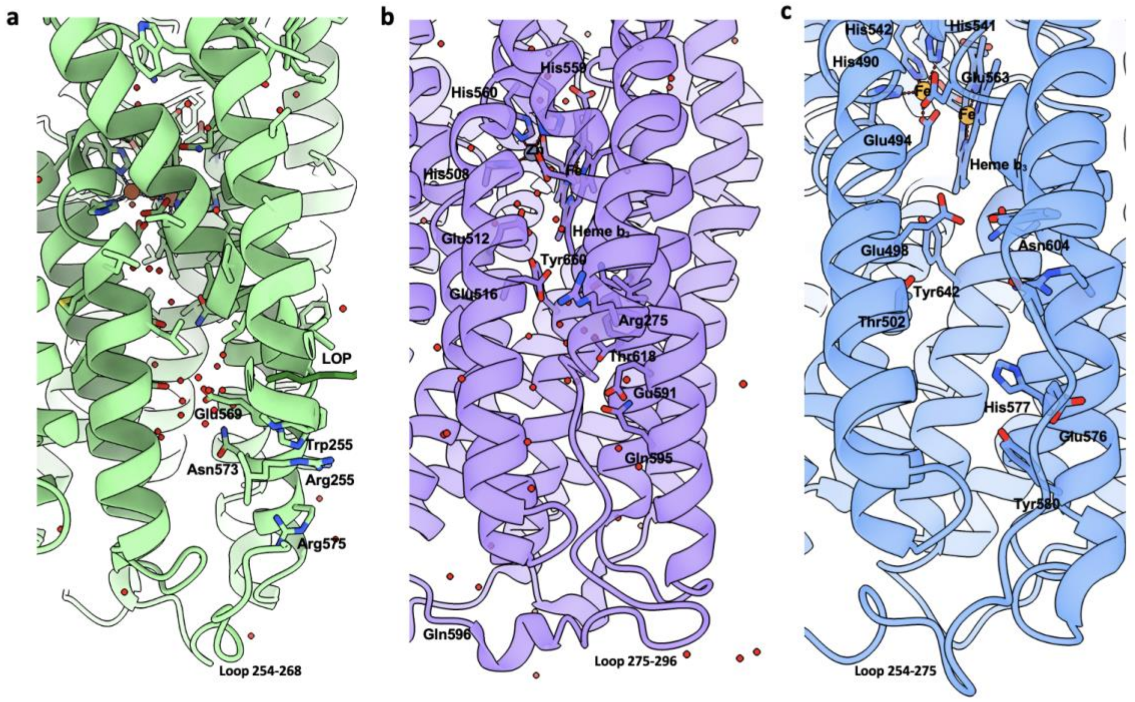


**Supplementary figure 2. Comparison of the cytoplasmic part of product release channels at the dimeric interface in structurally characterized qNORs.** (a) Product release path of qNOR from *Alcaligenes xylosoxidans* (*Ax*qNOR) from the cytoplasmic side to the binuclear centre. *Ax*qNOR coloured in green with residues lining the proposed path in *Ax*qNOR shown as sticks. Fe atoms and water molecules are shown as orange and red spheres, respectively. Hydrogen bonds and metal co-ordination bonds shown as black and red dashed lines, respectively. This scheme is used throughout the figure. (b) Product release channel in qNOR from *Geobacillus stearothermophilus* (*Gs*qNOR) (PDB ID: 3AYG) coloured in purple, with residues lining its proposed proton channel highlighted as sticks. Zn shown as grey sphere. (c) Product release channel in qNOR from *Neisseria meningitidis* (*Nm*qNOR) coloured in blue, with residues lining its proposed proton channel highlighted as sticks (PDB ID: 6L3H**).**

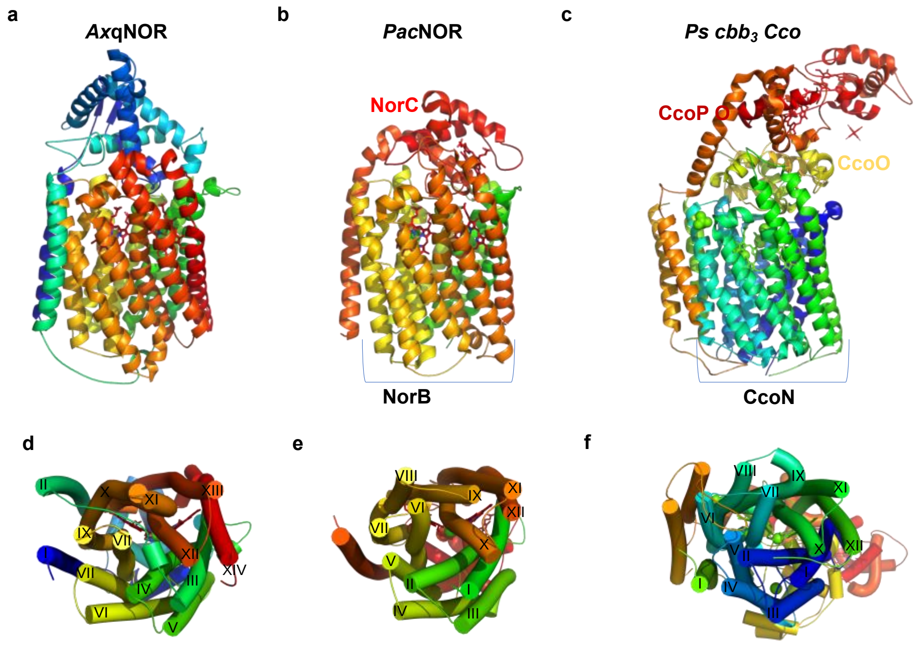


**Supplementary figure 3. Comparison of overall structures of *Axq*NOR, *Pac*NOR and *Ps* cbb$_3$ Cco.** (a) Overall structure of *Ax*qNOR viewed parallel to the membrane shown as ribbon in various colors; heme *b* and heme *b$_3$* are shown as red sticks. $Fe_B$ and calcium ions are represented as orange and green spheres, respectively. (b) Overall structure of cNOR from *P. aeruginosa* viewed parallel to the membrane (PDB code 3WFB). The NorB and NorC subunits are shown as ribbon in various colors and red, respectively. Heme groups are shown as red sticks. $Fe_B$ and calcium ions are represented as orange and greens spheres, respectively. Average rmsd between 390 C$\alpha$ atoms of *Ax*qNOR and NorB subunit is 4.3 Å; (c) Overall structure of Ps *cbb$_3$* cox (PDB code 5DJQ) viewed parallel to the membrane shown as ribbon in various colors for subunit N, in yellow for subunit O; and in red for subunit P. Hemes b and b$_3$ are shown as green sticks. (d) Arrangement of membrane-spanning helices of *Ax*qNOR viewed from the periplasmic side. Transmembrane helices are indicated with Roman numerals in all panels. (e) Arrangement of membrane-spanning helices of *Pac*NOR viewed from the periplasmic side. (f) Cytochrome cbb$_3$ oxidase from (PDB code 5DJQ). Subunit N with helices I ~ XII is shown in multi color. Average rmsd between 278 C$\alpha$ atoms of *Ax*qNOR and CoxN subunit is 8 Å Helixes of subunit O and helixes of subunit 3 are shown in yellow and orange-red, respectively.

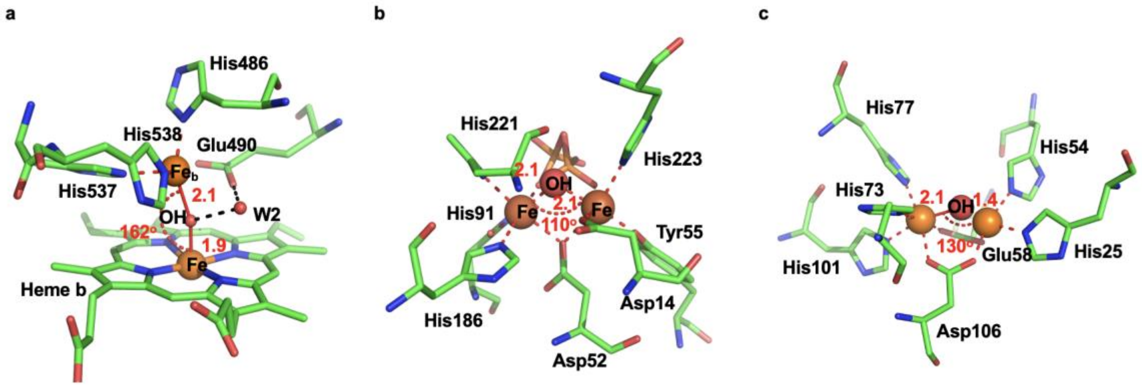


**Supplementary figure 4**. Di-iron sites with bridging hydroxide ion. (a) Fe bi-nuclear site of *Ax*qNOR. Non heme Fe is ligated by His486, His 537 and His 538 and OH ion with slightly distorted tetrahedral geometry. The hydroxide (OH) is bound in μ-oxo configuration between two metals at 2.1 Å distance to $Fe_B$ and 1.9 Å to heme Fe, the distance between two Fe is 3.9 Å. Angle between two metal bonds is 162°. (b) Fe bi-nuclear site of Pig tartrate-resistant acid phosphatase type 5 (PDB: 5UQ6 [https://doi.org/10.2210/pdb5UQ6/pdb]); the distance between two Fe is 3.4 Å. (c) Fe bi-nuclear site of human Hemerythrin (PDB: 2HMQ [https://doi.org/10.2210/pdb2HMQ/pdb]), the distance between two Fe is 3.2 Å. Residues are shown as sticks, Fe ions as orange spheres, water molecules and OH ion as red spheres.

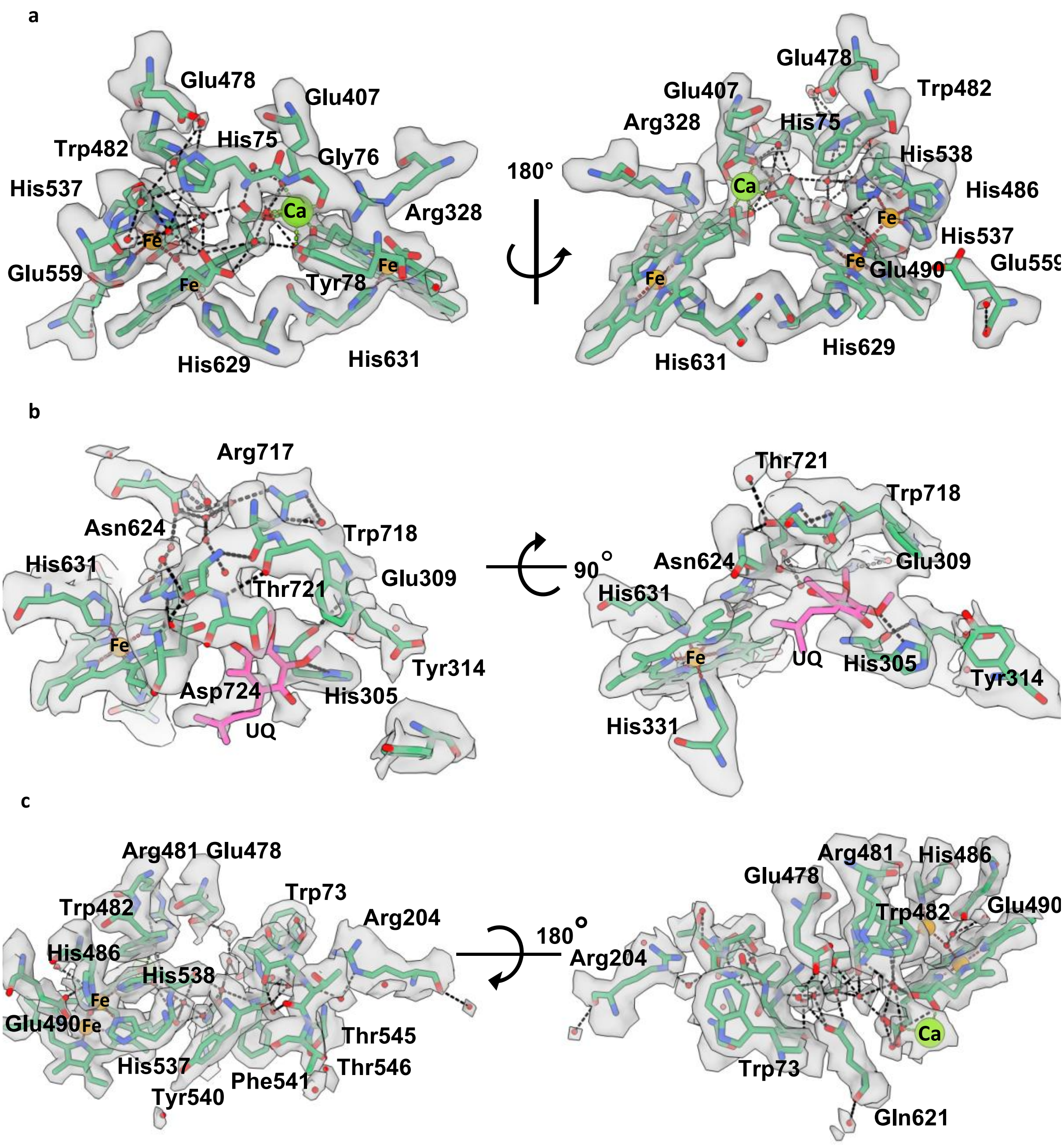


**Supplementary figure 5:** The cryo-EM map for key residues surrounding key elements and residues (a) *Axq*NOR calcium site. Cryo-EM map shown with threshold set to 0.023. (b) Key residues around the *Axq*NOR electron donor binding site. Ubiquinone electron donor shown as pink sticks. Cryo-EM map shown with threshold set to 0.1. (c) *Axq*NOR NO entry site and surrounding residues. Cryo-EM map shown with threshold set to 0.020. AxqNOR residues shown as green sticks. Cryo-EM map shown as a translucent grey surface. Waters, iron ions and calcium ions shown as red, orange and green spheres, respectively.

```
AxqNOR      -----------------MGPYRRLWFTLIAVLAVTFALLGFYGGEVYRQAPPIPEEVASA     43
GsqNOR      MEVNRTVSPNIQTGRKTTNSFLKS--ILIFTILISSTVLLVGGYWIFKEMAPRPKEVRSE     58
NmqNOR      -----------------MGQYKKLWYLLFAVLAVCFTILGYMGSEVYKKAPPYPEQVVSA     43
                              . : :    *: .: :  ::*   *  ::::  * *::* *

AxqNOR      DGTRLFGRDDILDGQTAWQSIGGMQLGSIWGHGAYQAPDWTADWLHRELMAWLDLAARDA     103
GsqNOR      SGEVLMTKETIIGGQAVFQKYGLMDYGTVLGHGSYMGPDYTAEALKVYTEGMQDYKAKER     118
NmqNOR      SGKVLMAKDDILAGQSAWQTTGGMEVGSVLGHGAYQAPDWTADWLHRELSAWLDLTAQQT     103
            .*  *: :: *: **:.:*. * *: *:: ***:* .**:**: *:    .  *  *::

AxqNOR      HGRDYGQLDAPAQAALREQLKAEYRANRADAAGGKLTLSPRRAQAVAQTEAYYDQLFSDA     163
GsqNOR      YNKPFADLTDDEKSIIREQVIKEMRKNRYNPVTDVLVLTDAQVYGLEKVRDYYRDVFTNG     178
NmqNOR      YGKKFDEVSPEEQAVLKTRLADEYRNQSRIKEDGSVVISDTRVKAIESILPYYHGVYGDD     163
            :.: : ::    :: :: ::  * * :      . :.::  :. .: .   **  :: :

AxqNOR      PALHRSRENYAMKENT--------LPDANRRRQMTHFFFWTAWAAGTEREGTSVTYTNNW     215
GsqNOR      DGWGLKKG--LIKESDMPKANRAWVADSDQIQQIADFFFWTAWLSSTLRIGDEITYTNNW     236
NmqNOR      PALQTTREHFAMKNNT--------LPSQEAREKLFDFFFWTSWSASTNRPDETFTYTNNW     215
             .   .:    :*:.         : . :  .:: .*****:* :.* * .  .******

AxqNOR      PHEPLIGNHPSSENVMWSIISVVVLLAGIGLLIWAWAFLR---GKEEDEPPAPARDPLTT     272
GsqNOR      PYYEDAGNTMSFSAVWWSGASVTILILFIGIILYVFYRYQLSMQEAYAEGKFPV-IDLRR     295
NmqNOR      PHEPLINNVPTTENYMWSFTSVVLLLMGIGLLMWGYSFLT---KHEEVE--VPTEDPISK     270
            *:    .*  : .   **  **.:*:  **:::: :        .   *   *.   :

AxqNOR      FALTPSQRALGKYLFLVVALFGFQVLLGGFTAHYTVEGQKFYGID------LSQWFPYSL     326
GsqNOR      QPLTPSQVKAGKYFVVVSALFFVQTMFGALLAHYYTEPDSFFGINW-----IYDILPFNI     350
NmqNOR      VQLTPSQKALGKYVFLTVALFVVQVLLGGLTAHYTVEGQGFYGIDEALGFEMSDWFPYAL     330
              *****   ***..:. *** .*.::*.: *** .* : *:**:      : : :*: :

AxqNOR      VRTWHIQSALFWIATGFLAAGLFLAPLINGGRDPKYQKAGVDILFWALVLVVVGSFAGNY     386
GsqNOR      AKGYHLQLAIFWIATAWLGMGIFIAPLV-GGQEPKKQGLLVDLLFWALVVLVGGSMIGQW     409
NmqNOR      TRTWHIQSAIFWIATGFLTAGLFLAPIVNGGKDPKFQRAGVNFLYIALFIVVGGSYAGNF     390
            .: :*:* *:*****.:*  *:*:**:: **::** *   *::*: **.::* **  *::

AxqNOR      LAIAQIMPPDLNFWLGHQGYEYVDLGRLWQIGKFAGICFWLVLMLRGIVPALRTPGGDKN     446
GsqNOR      LGVNGYLGNE-WFLLGHQGWEYIELGRIWQIILVVGMLLWLFIVFRGVKRGLKRESDKGG     468
NmqNOR      FALTHILPPEFNFWFGHQGYEYLDLGRFWQLLLMVGLLLWLFLMLRCTVSAFKEKGVDKN     450
            :.:   :  :  * :****:**::***:**:  ..*: :**.:::*    .::  . . .

AxqNOR      LLALLTASVGAIGLFYGAGFFYGERTHLTVMEYWRWWIVHLWVEGFFEVFATTALAFIFS     506
GsqNOR      LIHLLFYSAIAVPFFYIFAFFIQPDTNFTMADFWRWWIIHLWVEGIFEVFAVVVIGFLLV     528
NmqNOR      LLAIFVASMVGVGVFYAPGLFYGEKSPIAVMEYWRWWVVHLWVEGFFEVFATAAFAFVFY     510
            *: ::  *  .: .**  .:*    : ::: ::****::******:*****...:.*::

AxqNOR      TLGLVSRRMATTASLASASLFMLGGIPGTFHHLYFAGTTTPVMAVGASFSALEVVPLIVL     566
GsqNOR      QLRLVTKKSTVRALYFQFTILLGSGVIGIGHHYYNGSPEVWIALGAVFSALEVIPLTLL     588
NmqNOR      NMGFVRRSTATASTLAAAAIFMLGGVPGTLHHLYFSGSTSASMAIGACFSALEVVPLVLL     570
             : :* :  :. :     :::: .*: *  ** *: *:    :*:** ******:** :*

AxqNOR      GHEAWENWRLKTRAPWMENLKWPLMCFVAVAFWNMLGAGVFGFMINPPVSLYYIQGLNTT     626
GsqNOR      ILEAYEQYKMMRDGGANFPYKATFWFLISTAIWNLVGAGVFGFLINLPAVSYFEHGQFLT     648
NmqNOR      GREAYEHWSYQHLSEWAKRLRWPLMCFVAVAFWNMIGAGVFGFLINPPISLFYIQGLNTS     630
              **:*::     .      :  :  :::.*:**::*******:** *   :: :*   :

AxqNOR      PVHAHAALFGVYGFLALGFTLLVLRYIRPQYALSPGLMKLAFWGLNLGLALMIFTSLLPI     686
GsqNOR      PAHGHAAMMGVYGMFAIAVLLYSLRNIVKPEAWNDKWLKFSCWMLNIGLAGMVVITLLPV     708
NmqNOR      AVHAHAALFGVYGFLALGFVLLVARYLKPNVQFDDKLMTWGFWLLNGGLVGMIAISLLPV     690
             .*.***::****::*:.. *   * :      .   :. . * ** **. *:  :***:

AxqNOR      GLIQFHASVSEGMWYARSEAFMQQDILKTLRWGRTFGDVVFLLGALAMVVQVILGLLSGK     746
GsqNOR      GILQMKEAFIHGYWASRSPSFLQQDVVQNLLVRAVPDTIFLIGVVALLVFAIKALFHLR     768
NmqNOR      GVIQAYASITHGLWYARSEEFLQMEILDTLRWVRTAADLIFIGGAICVAIQATKIVFGRD     750
            *::*   :. .* * :**  *:* :::..*   *:  * :*: *.:.: : .   ::

AxqNOR      --------------------------746
GsqNOR      KPTHGEGEELPVANHWMKDRLKNSLE794
NmqNOR      K-------------------------751
```

NO entry channel; heme, $Fe_b$ ligation; Ubiquinol binding; Ca binding ligands

**Supplementary figure S6A. Sequence alignment of *Axq*NOR, *Nmq*NOR and *Gsq*NOR.** Homology between *Ax*qNOR (PDB:8BGW [https://doi.org/10.2210/pdb8BGW/pdb]) and *Nm*qNOR(PDB:6L3H [https://doi.org/10.2210/pdb6L3H/pdb]) is 58.7%; Homology of *Ax*qNOR and *Gs*qNOR is 33.3%. Residues or NO entry channel are highlighted in grey; heme Fe *b* and $F_B$ ligands in cyan; Ubiquinol binding site in magenta and Ca binding ligands in Green; fully conserved residues are marked with *. Alignment is performed by CLUSTRALW EBI server.

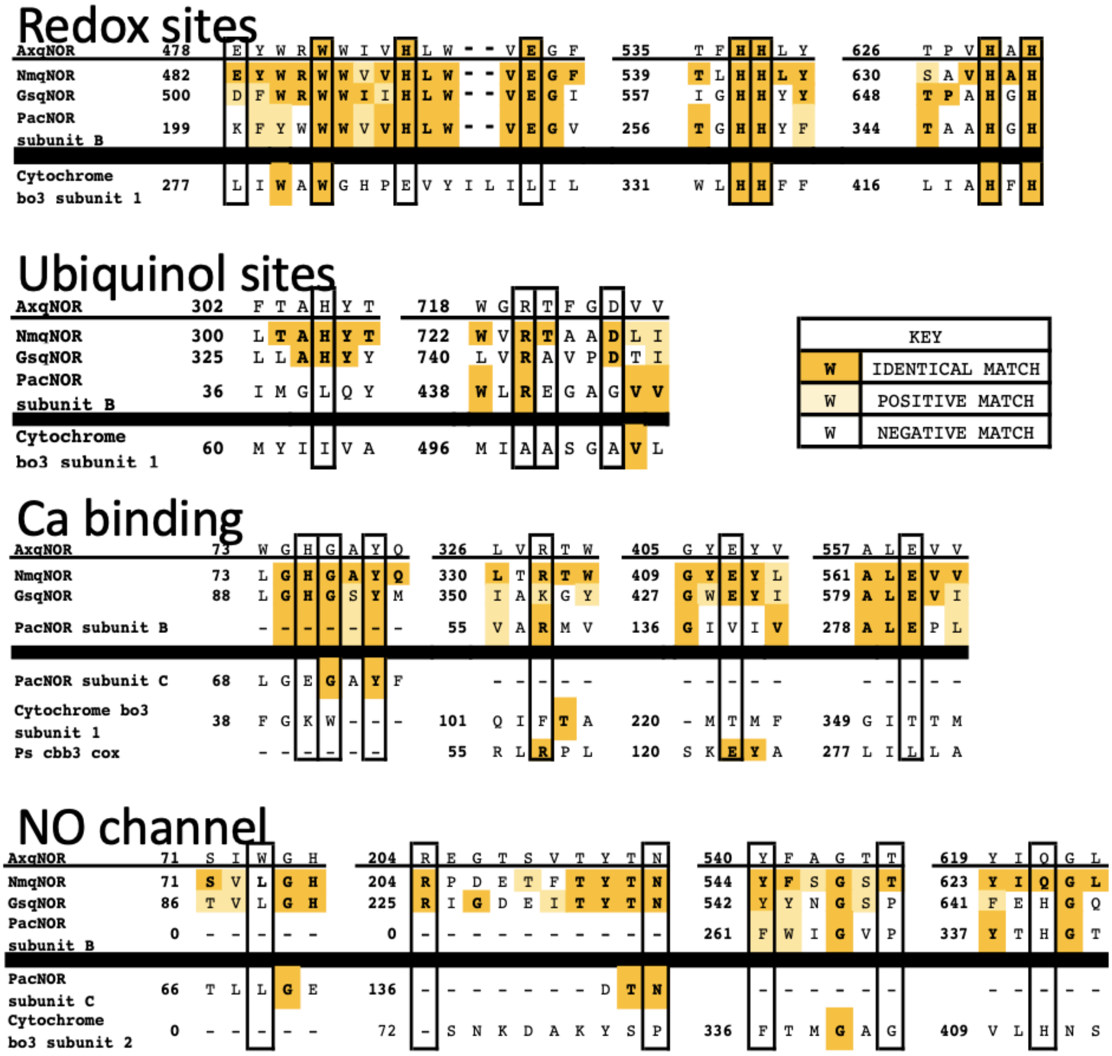


**Supplementary figure 6B. Sequence fragments conservation for*Axq*NOR, *Nmq*NOR, *Gsq*NOR, PacNOR subunits B and C and subunit 2 of COX.** Identical residues are colored in dark yellow, positive matches by light yellow. The upper sequences were aligned by pairwise alignment against *Axq*NOR sequence using BLAST and scored according to the BLOSUM62 score matrix. The bottom sequences were not similar enough to *Axq*NOR for pairwise alignment, so were aligned by multiple sequence alignment with the upper sequences using Clustal Omega. These aligned sequences were not scored by a matrix and so were only marked for identical residues.

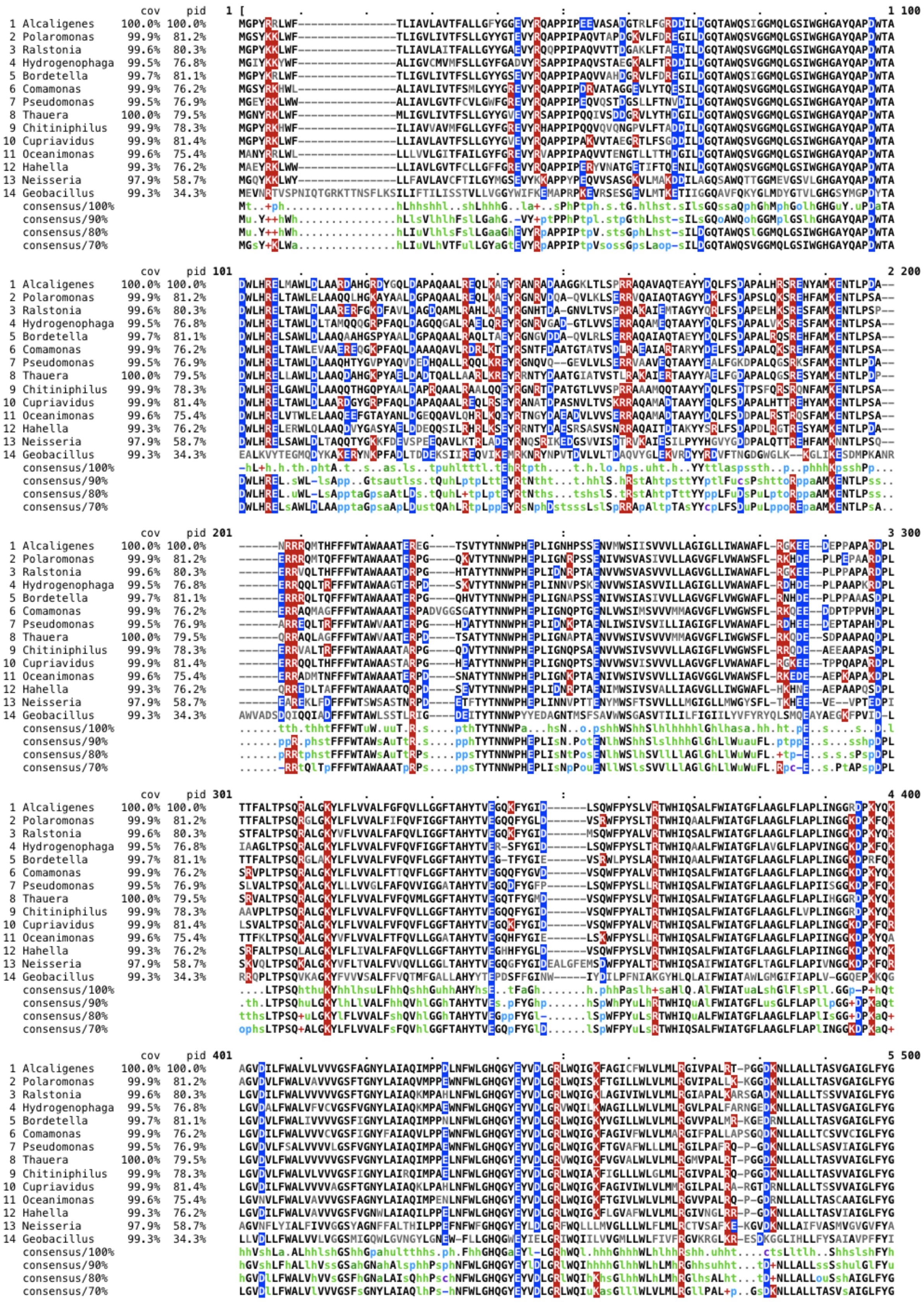

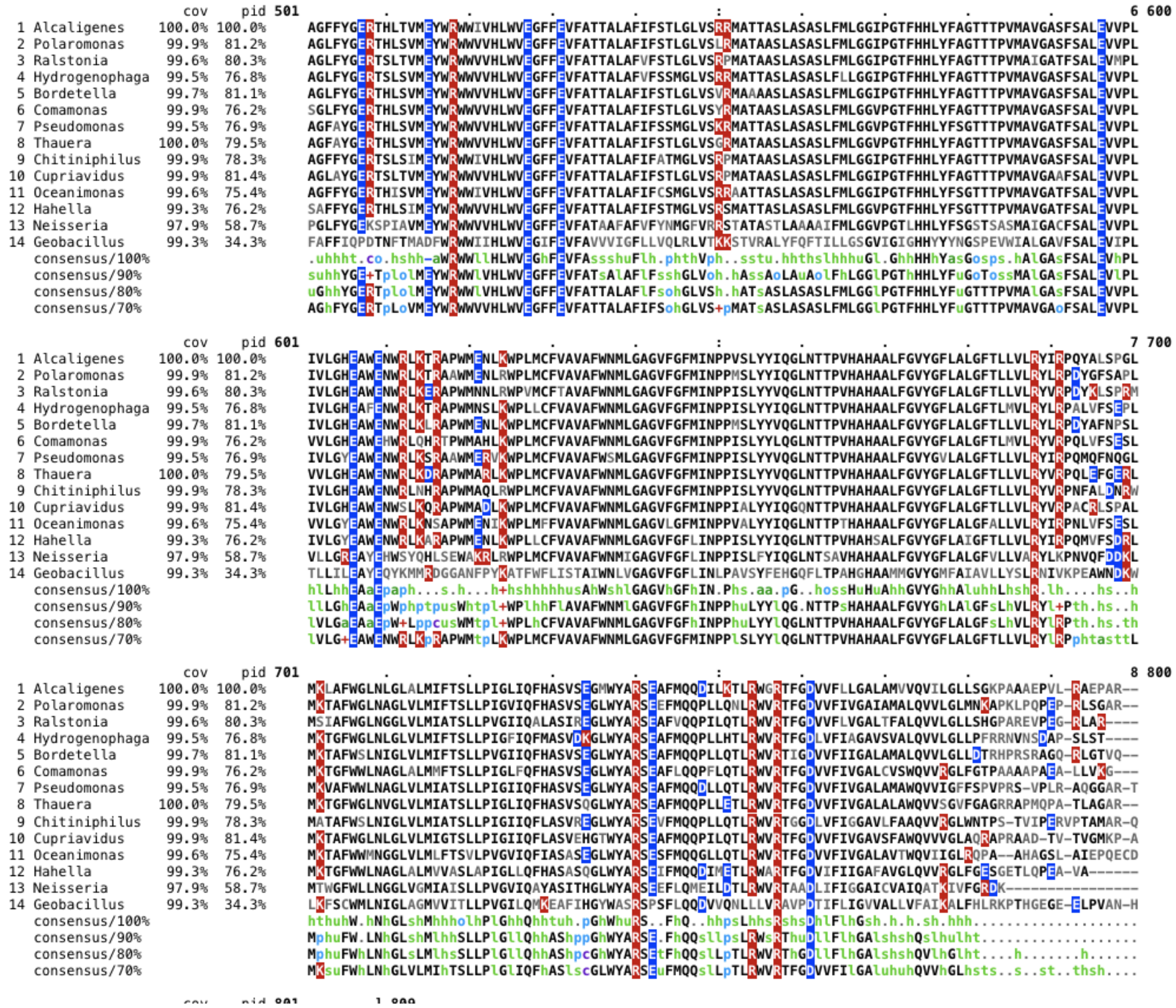

**Supplementary figure 6C. Sequence alignment of representative *Axq*NOR homologs**. *Alcaligenes xylosoxydans* (uniprot A0A0D6H8R3); *Polaromonas sp.* (A0A1H0FNK7); *Ralstonia sp.*( A0A1H0U9C7); *Hydrogenophaga intermedia* (A0A1L1PKZ3); *Bordetella genomosp* (A0A261SF53); *Comamonas terrigena* (A0A2A7UR79); *Thauera sp* (A0A2W6XYG8); *Pseudomonas putida* (A0A3M8T612); *Chitiniphilus eburneus* (A0A4U0PXB0); *Cupriavidus taiwanensis* ($B_3$RCN9); *Geobacillus stearothermophilus* ($B_3$Y963); *Oceanimonas sp.*(H2G1F4); *Hahella chejuensis* (Q2SP13).Consensus between residues is highlighted by % and color; % of identity between sequences is shown in “pid” column. Alignment is performed by CLUSTRALW EBI server.

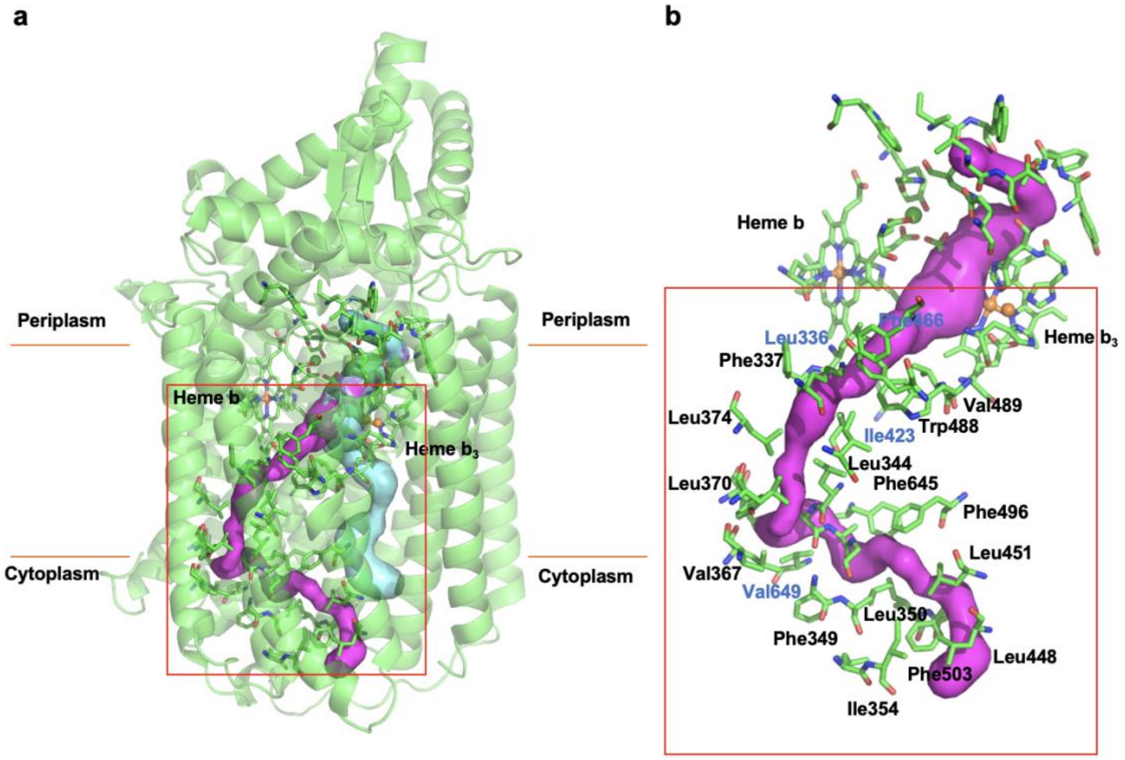


**Supplementary figure 7. Putative product release pathway in *Axq*NOR. (a**) Location of the putative product release path from binuclear active site to the cytoplasm is shown in purple and based on CAVER 3.0 analysis. Water channel is shown in cyan. (b) Hydrophobic residues surrounding the channel and heme molecules are shown as sticks. Iron and Ca ions are represented by orange and green spheres. Part of the channel directed to the cytoplasm is indicated by the red square. Majority of the residues lining the path are strictly conserved. Residues with black labels are strictly conserved; residues with blue labels are semiconserved.

.

**Supplementary Table 1: Cryo-EM data collection, refinement and validation statistics**

| | |
|---|---|
| **Data Accession** | |
| PDB | 8BGW |
| EMDB | 16041 |
| | |
| **Data Collection** | |
| Microscope | FEI Titan Krios |
| Voltage (kV) | 300 |
| Detector | Falcon 4 |
| Energy filter slit width (eV) | 10 |
| Nominal magnification | 130k |
| Pixel size (Å/pixel) | 0.91 |
| Defocus range (μm) | -0.9 - -2.7 |
| Exposure time | 6.11 |
| Frames | 44 |
| Exposure rate ($e^{-}$ $pixel^{-1}$ $s^{-1}$) | 4.61 |
| Electron exposure ($e^{-}$/Å$^{2}$) | 34.90 |
| Dose per frame ($e^{-}$/Å$^{2}$) | 0.8 |
| Micrographs collected | 5466 |
| **Reconstruction** | |
| Software | RELION 3.1 |
| Particles used in refinement | 404,950 |
| Symmetry | C2 |
| Overall resolution when FSC=0.143 (masked) (Å) | 2.2 |
| Map sharpening B-factor (Å$^{2}$) | -47.68 |
| | |
| **Model Refinement** | |
| Software | REFMAC5 |
| Non-hydrogen atoms | 12845 |
| Protein residues | 11649 |
| Ligands | 746 |
| Average B factors (Å$^{2}$) | 61.128 |
| Protein | 63.29 |
| Ligands and water | 91.95 |
| R.M.S. deviations | |
| Bond length (Å) | 0.012 |
| Bond angle (Å) | 1.649 |
| Ramachandran statistics (%) | |
| Outliers | 0.27 |
| Allowed | 5.47 |
| Favoured | 94.26 |
| MolProbity score | 2.36 |
| ClashScore | 15.19 |
| Poor rotamers (%) | 2.41 |
| Model vs. Map FSC | 0.854 |

**Supplementary Table 2: Comparison of Ca ligands and bond distances in *Axq*NOR, its mutant and *Pac*NOR.**

| Ca ligands in *Axq*NOR (*Pac*NOR) | 2.2 Å *Axq*NOR<br><br>**8BGW** | 3.9 Å AxqNOR<br><br>**6QQ5** | 3.3 Å AxqNOR Variant<br>**6QQ6** | 2.7 Å *Pac*NOR<br><br>**3O0R** |
|---|---|---|---|---|
| Water | 2.3 | - | - | 2.1 |
| OH Tyr78(73*) | 2.5 | 2.6 | 2.7 | 2.6 |
| O Gly76(71*) | 2.3 | 2.4 | 2.8 | 2.3 |
| O2A Heme $b_3$ | 2.3 | 2.6 | 2.1 | 2.6 |
| O1D Heme b | 2.2 | 2.3 | 2.5 | 2.4 |
| O2D Heme b | (3.7) | 2.7 | 2.5 | (3.8) |
| OE2 Glu407(135) | 2.1 | 2.5 | 2.4 | 2.8 |
| OE1 Glu407(135) | 2.2 | 2.9 | 3.0 | 2.6 |
| * residues numbers for *Pac*NOR | | | | |
